# Primary Cilia–An Underexplored Topic in Major Mental Illness

**DOI:** 10.3389/fpsyt.2019.00104

**Published:** 2019-03-04

**Authors:** Michal Pruski, Bing Lang

**Affiliations:** ^1^Department of Psychiatry, The Second Xiangya Hospital, Central South University, Changsha, China; ^2^Critical Care Laboratory, Critical Care Directorate, Manchester Royal Infirmary, Manchester University NHS Foundation Trust, Manchester, United Kingdom; ^3^School of Healthcare Science, Faculty of Science and Engineering, Manchester Metropolitan University, Manchester, United Kingdom; ^4^School of Medicine, Medical Sciences and Nutrition, Institute of Medical Sciences, University of Aberdeen, Aberdeen, United Kingdom

**Keywords:** primary cilia, schizophrenia, bipolar disorder, neurodevelopment, brain

## Abstract

Though much progress has been made in recent years towards understanding the function and physiology of primary cilia, they remain a somewhat elusive organelle. Some studies have explored the role of primary cilia in the developing nervous system, and their dysfunction has been linked with several neurosensory deficits. Yet, very little has been written on their potential role in psychiatric disorders. This article provides an overview of some of the functions of primary cilia in signalling pathways, and demonstrates that they are a worthy candidate in psychiatric research. The links between primary cilia and major mental illness have been demonstrated to exist at several levels, spanning genetics, signalling pathways, and pharmacology as well as cell division and migration. The primary focus of this review is on the sensory role of the primary cilium and the neurodevelopmental hypothesis of psychiatric disease. As such, the primary cilium is demonstrated to be a key link between the cellular environment and cell behaviour, and hence of key importance in the considerations of the nature and nurture debate in psychiatric research.

## Introduction

Recently Muñoz-Estrada et al. (1) published results from experiments on olfactory neuronal precursor cells obtained from human sufferers of schizophrenia (SCZ) and bipolar disorder (BD), linking primary cilia (PC) with major mental illness (MMI). Their work showed a general decrease in the percentage of cells with PC in subjects suffering from MMI. Furthermore, *in vitro* supplementation with lithium (a common pharmacotherapy for BD, mania and depression (2), and previously shown (3) to cause *in vivo* and *in vitro* PC elongation in mouse neuronal cells) was shown to have a positive effect on PC length. While their study (1) was conducted on samples obtained from a very limited number of patients suffering from a variety of MMI and on different treatment regimes, it highlights an area of psychiatric research that has been largely ignored.

PC are cellular protrusions originating from the centrosome's mother centriole, and are present on most mammalian cells (4, 5). Since they are linked with the centrosome, they need to be disassembled or retracted whenever the centrosome needs to perform its microtubule organizing centre functions, such as during cell division and migration (4, 6–9), making the exact role of the PC in these processes somewhat unclear. PC are largely regarded as cellular sensory antennae and signalling hubs, facilitating key developmental pathways such as Sonic Hedgehog (SHH) and WNT signalling (10–12). More recently, proposals have been made that PC have an extracellular signalling role, as thanks to its biochemical autonomy from the rest of the cell membrane the cilium can express distinct proteins on its membrane as well as have a different concentration of various factors in its cytoplasm (13, 14). As such ciliary vesicles, can form distinct exosomic parcels, but their role, especially in mammals, is not yet clear, and arguments have been made that their primary function is to dispose of redundant ciliary content (13, 14). This article reviews various levels of evidence for the role of PC in MMI, focusing on the well-established developmental hypothesis of MMI (15, 16). PC play an important role in the development of the central nervous system (CNS) and in a wide variety of roles in the adult brain (10, 17). PC have been even called “neurons little helpers” in the context of neurodevelopment (18), and have recently been discussed in the context of the neuronal migration hypothesis of dyslexia (19). Nevertheless, as discussed later, PC retract during some cellular events, and might not be present on all types of neurons during early development (8, 20). The following sections will show why this elusive organelle should be considered an attractive target for psychiatric research.

## Genetics

Genomic and bioinformatics research has revealed that some PC genes are linked to MMI. Of course, the mere fact that a gene might be linked to both PC and MMI does not guarantee the involvement of PC in MMI, as proteins might function in different cellular compartments and in different cellular processes. Similarly, a different severity of mutation might be required to cause an effect on the PC or to precipitate MMI. Nevertheless, the fact that data from an RNA interference study looking at 41 MMI genes found that 23 affect cilia length (21) should at least prompt one to look at the correlation between PC and MMI genes. Here, we present evidence for two candidate genes which may potentially connect PC with MMI.

Previously (22), researchers described a region on chromosome 4p linked to MMI. One gene found in that region was CC2D2A (23), also known as MKS6 and JBTS9. It is involved in ciliogenesis (24), and in vesicles trafficking in the PC's transition zone (the region of the cilium that regulates the trafficking of proteins between the cilium and the rest of the cell, allowing the cilium to retain its distinct protein composition), and has been implicated in neural tube development and Sonic Hedgehog signalling (25–27). CC2D2A has been linked to a range of CNS developmental conditions linked to PC: Joubert syndrome (28–30), Meckel syndrome (31) and mental retardation (32, 33); there is also a potential link with Bardet-Biedl syndrome (BBS) (29). CC2D2A's link to MMI has not yet been thoroughly investigated (34), but MMI problems have been observed in individuals with Joubert syndrome, and AHI, also associated with Joubert syndrome, has been proposed as a marker for SCZ (35).

Disc1, a gene involved in the formation and regulation of cilia (36), has also been associated with MMI (37, 38). DISC1 associates with a variety of centrosomal components (39, 40), recruiting BBS proteins to the centrosome (41, 42), and acting as a switch between the processes of neuronal migration and proliferation. DISC1 also interacts with the dynein complex (43), which, together with the Intraflagellar Transport (IFT) complex A, is vital for retrograde transport within the PC (12). Moreover, one reported zebrafish DISC1 aberration caused a decrease in β-catenin levels (44) correlating to a decrease in canonical WNT activity.

As such, DISC1 is perhaps one of the strongest links between PC and MMI.

## Neurodevelopment

PC associated CNS defects range from cerebellar hypoplasia through mental retardation to encephalocele and enlarged ventricles (45–47). Moreover, various neurodevelopmental defects have been associated with MMI, for which there is evidence of PC involvement. This is unsurprising, as PC are present from the earliest stages of CNS development through to the mature brain (17, 48, 49). The centrosome, with which PC are closely interlinked, is also a key player in CNS development (50, 51).

Defective neuronal migration has been reported in several studies relating to MMI, and is likely to contribute to reductions of grey matter in patients affected by MMI (15, 52–56). Additionally, BBS has been associated with cortical volume reductions in both a human and mouse study (57, 58). Molecular links between psychiatric pathways and PC, such as the interaction of DISC1, WNT signalling, the BBS complex and the centrosome exist in the context of cell migration and proliferation (41–43, 59, 60). For example, PC have been shown to be involved in several aspects of neuronal migration like radial glial scaffold formation and interneuron migration (8, 61) as well as galvanotaxic migration (62–64).

PC factors also influence important migratory processes of microtubule (65–67) and actin (68, 69) organisation. Further, CDC42, a molecule important for ciliary initiation (70–72) promotes actin skeleton remodelling (73) and cell polarity (74, 75) through non-canonical WNT signalling (51). Furthermore, CDC42 and actin skeleton remodelling have been associated with deficits in dendritic spine formation frequently reported in SCZ and BD (76–78). Issues relating to neuronal network health, such as synaptic connectivity and neurite number have also been highlighted in both mental illness and PC dysfunction (53, 54, 79–85).

Problems with neuronal differentiation have been associated with SCZ, and can result from DISC1 related changes in WNT signalling (86–88). Asymmetric PC membrane inheritance occurs during neocortical development, and is linked with the inheritance of the centrosome, which is important for proper neurogenesis (89–91), suggesting that PC function might be important for cell division and fate specification, further contributing to the aforementioned changes in cortical volume (57, 58).

Moreover, PC are known to be involved in other developmental aspects that could contribute to defective neurogenesis and CNS cell migration, often involving PC's close association with the centrosome and the Golgi apparatus. These involve SHH (92–94) and Platelet Derived Growth Factor (95–98) signalling, and governance of cell migration (4, 8, 9, 50, 63, 99–102) and cell division (95–97, 103–110) through sensing extracellular cues. This last point is exemplified by the fact that serum withdrawal during cell culture is a ciliogenic condition (97, 103, 105, 109), indicating that cells may use their cilia to ensure that the extracellular conditions are right for mitosis initiation.

## WNT Signalling

The WNT pathway is one of the best studied MMI signalling pathways (111–114). Those affected by BD and SCZ have been found to express mRNA levels suggestive of attenuated canonical WNT signalling and enhanced non-canonical signalling, particularly the WNT/Ca^2+^ pathway (111); although a recent human cerebral organoid study showed an increase in canonical WNT signalling in the early developmental stages of brains with disrupted DISC1, suggesting that WNT changes might be context/age dependent (114). The changes in mRNA expression levels is a noteworthy finding since PC are known to facilitate the switch from canonical to non-canonical WNT signalling via Ca^2+^ signalling (103). PC modulates WNT signalling via the degradation of Dishevelled by Inversin at the basal body (51, 115, 116), repressing the canonical signalling pathway (117, 118) and promoting the planar cell polarity pathway (95). Curiously, different ciliary gene mutations perturb WNT signalling in different ways, with mutations in some genes being able to both increase and decrease β-catenin levels (119).

Moreover, WNT signalling affects motile cilia, and might affect PC by influencing basal body positioning on the apical membrane (51, 120–122). The importance of such an overlap and interaction between motile and primary cilia has been highlighted in hydrocephalus (96), where defects in both motile and primary cilia are known to be present (96, 123). Hydrocephalus-like changes have similarly been reported in SCZ (124). Motile cilia generate fluid flow, to which PC respond (125), which is crucial for establishing body asymmetry (126), and is detected by the polycystin receptors PC1 and PC2 (127–129), which facilitate Ca^2+^ entry (127). This flow-induced calcium signalling not only facilitates the switch from canonical to non-canonical WNT signalling (103) but also regulates the cell cycle (95), although recent experiments have started questioning whether flow sensing happens via Ca^2+^ signalling (130).

Nevertheless, there is some evidence disputing PC's role in WNT signalling (11, 119). There is evidence from both zebrafish and mice showing that disrupting PC does not affect WNT signalling (131–133). The role of PC in WNT signalling is further complicated by the fact that WNT signalling has a regulatory role in ciliogenesis (120, 134, 135). As such, the exact role of PC in WNT signalling, particularly in canonical WNT (11), requires further investigation, though as argued in this section, such an investigation might bring fruitful results if carried out in the MMI context.

## Fibroblast Growth Factor

The importance of the Fibroblast Growth Factor (FGF) signalling system has been highlighted in SCZ research (136–138). This system can regulate neuronal differentiation via the Stat1 pathway, and neuronal proliferation and function via the ERK pathway (139). Moreover, FGF function has a positive effect on dopamine neuron survival and neurite outgrowth (139). Recently, bioinformatic and stem cell experiments investigated the role of the FGF receptor 1 (FGFR1) (136, 137). FGFR1 dysregulation can upregulate developmental pathways involved in neurogenesis and downregulate those involved in oligodendrogenesis (136), and data suggests that it can also lead to cortical maldevelopment (137), though the dysregulation of this pathway still awaits confirmation in a larger patient sample.

PC themselves do not seem to mediate FGF signalling, yet both motile and tethering cilia (a type of kinocilium, located on hair cells in the ear, with a microtubule structure similar to motile cilia (5, 140, 141)) length is affected by FGF (142–144). While it remains to be seen how ciliogenesis and PC length can be controlled by FGF in mammals, zebrafish and *Xenopus* studies suggest that FGF modulate the expression of Ift88 via FGFR1 (144). The IFT machinery is responsible for trafficking anterograde and retrograde cargo along the PC (145, 146) and IFT dysregulation can result in underdevelopment of certain organs, including the brain (145, 147). While the role of IFT in MMI requires further study, IFT27 [which together with IFT88 and IFT172 belongs to the IFT complex B (148)] has in one study been associated with BD (149), however the authors of that study note that this conclusion should be taken with caution due to the amount of variation present throughout the study. Since IFT172 has been identified as also being BBS20 (150) it is worthy to highlight that BBS is associated with such traits as reductions in hippocampal, white and grey matter volumes (57), traits often associated with MMI (151–154) and depression (155) belongs to the IFT complex B.

Therefore, if FGFR1 is proven to be implicated in a larger cohort of individuals with schizophrenia and in the regulation of human PC length, then there would be a mechanistic correlation between defective PC and SCZ. However, it would remain to be seen whether it was the PC dysfunction that contributed to SCZ or whether they were independent consequences of aberrant FGF signalling belongs to the IFT complex B.

## Primary Cilia and Dopamine

The dopamine hypothesis is prominent in SCZ research (156, 157), and various dopamine receptors localise to PC (36) in a manner dependent on IFT and BBS components (158, 159). Type 1 and 2 receptors have been shown to localise to PC in neurons in regions such as the striatum, amygdala, and pituitary gland (36, 158, 160, 161). Type 5 receptors, mediating both chemical and mechanical signalling in the PC, were shown on mouse endothelial cell (162), and type 4 receptors have also been shown on non-neuronal cells (36).

While the relationship of dopamine signalling, PC and MMI has not been explored, there might be an overlap between these during brain development. A possible explanation involves the dysregulation of WNT signalling important for the appropriate differentiation of dopaminergic neurons (163). The WNT pathway also regulates dopaminergic neural progenitor cell migration during electrotaxis (62); the health of PC has been shown to affect electrotaxis in fibroblasts (63), though studies in neurons are lacking. Moreover, dopamine signalling has been found to affect PC length in striatal neurons (160). As such, the implications of this interplay between PC and dopamine on MMI remain to be explored.

## Cilia–Nature and Nurture

The theme of this research topic compilation concerns neuropsychiatric disorders within the nature and nurture debate, and as such it is fitting to discuss how PC might fit within this debate. It is therefore valuable to reassess some of the aforementioned points within the context of some of the hypotheses of MMI.

The watershed hypothesis of MMI (164) suggests that the diseases might manifest themselves as the cumulative effects of smaller (potentially benign on their own) changes in physiological processes. PC dysfunction might contribute to small changes in several neurally important signalling pathways, not all of which have been mentioned here (165). These changes do not need to originate from serious mutations affecting a single gene (e.g., Disc1), but in themselves might be the result of several less severe changes in PC genes. Nevertheless, it must be remembered that some ciliary proteins might perform the majority of their work outside of the PC (166).

More importantly, genetic changes might, in themselves, not result in a pathological phenotype, but an environmental insult (or several) might be required to trigger the pathological process. This is known as the Two-Hit Hypothesis (167), and is of particular interest here due to the sensory role of the PC. There is a correlation between famine and SCZ (168–170), and there is experimental evidence that environmental stressors, such as maternal ethanol consumption, methylmercury exposure, and pentylenetetrazole (PTZ)-induced maternal seizures can cause neural damage to developing embryos, even at relatively low doses (171). This neural damage is associated with Heat Shock Factor expression level variability, which might be caused by oxidative stress damage (171). PC are involved in stress regulating pathways, such as ERK, but are also affected by the ERK response to oxidative stress and ischaemia (172, 173). Heat shock itself was found to cause ciliary absorption mediated via a reduced association of heat shock protein 90 with HDAC6, and was hypothesised to decrease PC mediated signalling during times of extracellular stress (174). Therefore, PC might provide a molecular link bridging the genetic and environmental components of MMI pathology.

## Future Directions

As noted, this manuscript explored several possible links between PC and MMI. Nevertheless, there is little literature directly exploring this topic. As such, we hope that this manuscript will encourage more research in this area. This section highlights some avenues that might be taken in this exploration.

PC length and frequency could be explored in histological specimens from animal models of MMI, and from human MMI patients. With the advances in microscopy and image analysis techniques [we have ourselves proposed such an analysis algorithm (175) for PC length], this is becoming a viable experimental strategy. Perhaps the biggest obstacle might be obtaining human post-mortem samples that would be of good enough quality to visualise PC.

This obstacle could be partly eliminated through the use of induced pluripotent stem cell technology, where human neurons (or other CNS cells) could be generated from tissues samples of MMI patients that could be ethically obtained during their lifetime. These cells could be subjected through a battery of tests, such as the study of their migration responses to a variety of cues. Such experiments would help to overcome several limitations highlighted in the text, e.g., the study of MMI and PC deficient neurons in electric fields. Moreover, using genetic editing technologies the effect of specific MMI associated mutations can also be investigated. These systems could also be used to evaluate the effects of environmental stressors on PC in MMI neurons, a link hypothesised in the previous section. The development of methods for growing cerebral organoids (114, 137), while perhaps raising ethical considerations, will allow for even more complex PC functions to be evaluated in a CNS-like environment.

Finally, while this paper has shown the involvement of PC in a range of signalling pathways. Yet, the evidence might not yet be strong enough to call the PC a signalling hub crucial for MMI. More research should be done to elucidate the role of PC in such key signalling processes for MMI as the dopamine and serotonin pathways (176) [5-HT6 receptors are predominantly expressed on PC (177, 178)], or to look at PC facilitation of pathways involved in the neurodevelopmental defects exhibited by those affected by MMI.

## Summary

This review has outlined why PC should be considered as an interesting area for MMI research (see summary in Table 1). It has demonstrated the involvement of PC in a wide variety of cellular processes, such as cell migration and proliferation, and as a signalling hub for various intracellular pathways related to MMI. PC have a unique ability to integrate information necessary for various developmental processes, and as such might be the missing link between the genetic and environmental causes of MMI.

**Table 1:** Summary of the key points from each section, and avenues for future research related to each section.

**Section**	**Key points**	**Future work**
Genetics	There is an overlap between genes associated with MMI and PC. Disc1 is the gene with the strongest connection to both MMI and PC.	The extent to which PC genes are associated with MMI requires further study via GWAS. The large amount of genes associated with PC can be both a source of false positive (due to pure statistical chance) and negative (watershed hypothesis, or small frequencies of any one particular gene or SNP) results. Identified genes should also have a mechanistic link between MMI and PC before a role of PC in MMI can be deemed conclusive.
Neurodevelopment	PC are involved in a range of developmental processes, such as cell migration and proliferation. Defects in these processes are associated with MMI.	Developmental processes can be disrupted in a variety of ways, as processes such as cell migration and proliferation depend on a variety components. Moreover, a single protein might act at several cellular locations. It is important that defects in ciliary proteins that are found to play a role in MMI, do this in a way that is mechanistically related to the PC. Additionally, changes in brain PC should be studied via histological samples from both well-established MMI animal models, and post-mortem patient brains.
WNT signalling	WNT is a major signalling pathway that has been implicated in MMI. PC have been often presented as providing a switch mechanism for the different modalities of WNT signalling.	Direct evidence of PC role in MMI WNT aberrations is still lacking. As such, iPSC studies should look at WNT signalling changes in MMI patients, and assess if any changes are due to changes in PC function.
Fibroblast growth factor	FGF signalling has been highlighted in SCZ. FGF affects expression of Ift88, a component of the ciliary transport machinery.	The interplay between FGF, MMI and PC is still poorly understood. As such, the avenues for exploration are very wide.
Primary cilia and dopamine	Dopamine signalling has been of major interest in SCZ research. Several dopamine receptors have been found on PC, including neuronal PC. Moreover, dopamine signalling has been found to affect ciliary length.	The importance of dopamine signalling via PC remains to be explored in the context of MMI, iPSC experiments from patient samples could be of great help here. This should be explored in both the contexts of adult brain function, and neurodevelopment.
Cilia–nature and nurture	PC's main function is to receive extracellular signals, and as such defects in PC can cause cellular defects in responding to extracellular cues. PC presents a key point of interaction between nature and nurture.	This is a complex and exciting area, as we grow in appreciation of the interactions between genes and the environment. Investigators would need to both assess whether some PC defects predispose people to aberrant reaction to environmental stressors, and whether some mutations, while not disrupting PC function in a healthy environment, might cause PC defects, resulting in neurodevelopmental defects, when exposed to environmental stressors.

PC and neuropsychiatric disorders are interesting fields for research, and much remains to be uncovered. While the arguments presented here show a correlation between PC and several different levels of biological processes associated with psychiatric disease as well as treatment, much yet remains to be experimentally proven. It is up to basic and clinical scientists to determine whether these are just correlations or if there is, indeed, a causative relationship between PC and MMI.

## Author Contributions

BL conceived the idea. MP wrote the manuscript with the support of BL.

### Conflict of Interest Statement

MP has been a student of two of the special issue editors (BL and YD), and as such collaborated with them on several projects in the last 5 years. BL is an editor of this special issue. All steps were taken to ensure impartial peer-review, and BL did not participate in making the decision about final acceptance of the manuscript for publication.
